# A Unique Case Report of *Leclercia adecarboxylata* Causing Monomicrobial Neurosurgical Infections

**DOI:** 10.1155/crdi/7844377

**Published:** 2025-10-14

**Authors:** Madeleine Purcell, Max Jacobs, Nazary Nebeluk, James B. Doub

**Affiliations:** ^1^Department of Internal Medicine, University of Maryland School of Medicine, Baltimore, Maryland, USA; ^2^Division of Infectious Diseases, University of Maryland School of Medicine, Baltimore, Maryland, USA

**Keywords:** emerging pathogen, Enterobacteriaceae, *Leclercia adecarboxylata*, spinal infection, ventriculitis

## Abstract

*Leclercia adecarboxylata* is an emerging gram-negative pathogen. Historically, it has primarily affected immunocompromised individuals and has mainly been isolated from polymicrobial infections. Yet, advances in diagnostic techniques have led to increased recognition of its incidence and clinical relevance. In this case series, two instances of monomicrobial *L. adecarboxylata* infections following neurosurgical procedures in immunocompetent patients are presented. The first case occurred after spinal laminectomy, and the other case caused external ventricular drain ventriculitis. Both patients responded well to targeted antibiotic therapy, with no sequela or recurrence of infection. These cases represent the first documented neurosurgical infections caused by *L. adecarboxylata*, highlighting the organism's potential as a nosocomial pathogen and underscoring the need for heightened clinical awareness and further investigation into its epidemiology to prevent further infections.

## 1. Introduction

*Leclercia adecarboxylata*, formerly*Escherichia adecarboxylata*, is a motile gram-negative rod belonging to the Enterobacteriaceae family. This organism resides in aquatic environments and soil and is part of the intestinal microbiome of mammals [1]. It has historically been considered a low virulence pathogen mainly causing disease in immunocompromised hosts [2]. However, since its discovery in 1962, *L. adecarboxylata* has steadily been increasing in recognition as a pathogen of clinical concern.

The most comprehensive review of *L. adecarboxylata* infections documented 74 cases in immunocompromised hosts, which included endocarditis, bacteremia, cellulitis, surgical site infections, pneumonia, and surgical hardware infections [3]. While most infections have been found in immunocompromised adults, there have been some cases of *L. adecarboxylata* infections in immunocompetent children and adults [4, 5]. Moreover, *L. adecarboxylata* is commonly isolated from polymicrobial infections but rarely causes monomicrobial infections [3, 6]. Here, we present a case series of two immunocompetent neurosurgical patients, who had monomicrobial *L. adecarboxylata* infections.

## 2. Case Reports

### 2.1. Case 1

A sixty-one-year-old female who underwent an elective L4-L5 laminectomy due to spinal stenosis presented 2 weeks after surgery with progressive headaches, clear drainage from her lumbar incision, and intermittent left leg weakness. MRI revealed a pseudomeningocele, and she underwent urgent neurosurgical evacuation. Operative findings did not show any breakdown of fascia but noted 50–60 cc of clear fluid consistent with cerebrospinal fluid. No overt purulence was observed, and intraoperative cultures did not isolate any pathogens.

One week later, she noted purulent drainage from the surgical wound without associated fevers or meningeal signs. Drainage progressed despite short courses of amoxicillin–clavulanic acid and azithromycin prescribed for what was thought to be an unrelated sinusitis. She returned to the hospital and underwent incision and drainage. Intraoperative findings showed cloudy yellow fluid without breakdown of the dura. Aerobic and anaerobic cultures were taken at the time of surgery. Gram stain showed abundant polymorphonuclear cells (PMNs) without organisms. Aerobic cultures from deep lumbar spinal fluid grew *L. adecarboxylata* with sensitivities, as shown in Table 1. She was initially treated with piperacillin–tazobactam but discharged on ceftriaxone 2 g IV daily for a 6-week course after anaerobic cultures resulted in no growth. Her C-reactive protein (CRP) decreased from 1.8 to 0.1 mg/dL by the end of the treatment course. Concurrently, her erythrocyte sedimentation rate (ESR) decreased from 80 to 15 mm/hour. The patient did not have any retained hardware, so prolonged antibiotic suppression was not considered. Four years posttherapy, the patient is without any evidence of recurrent or persistent infection.

### 2.2. Case 2

A forty-nine-year-old female with hypertension presented to the emergency department with severe headaches. A computer tomography (CT) scan of the head showed a ruptured anterior communicating artery aneurysm with diffuse subarachnoid hemorrhage and intraventricular hemorrhage. She underwent balloon-assisted coiling of the aneurysm with external ventricular drain (EVD) placement. A week later, the patient had an isolated fever of 39.6°C associated with tachycardia and hypoxia, requiring high-flow nasal cannula. A CT angiography scan of the chest was performed, which showed multifocal pneumonia involving the bilateral lower lobes. She was treated with a 7-day course of vancomycin and piperacillin–tazobactam. Twenty-four hours after completing antibiotic therapy, she became febrile with altered mental status.

On exam, a crack in her EVD was noted which prompted EVD exchange. Prior to exchange, a sample of CSF was collected (Table 2) based on which the patient was started on vancomycin and cefepime. Over the next 4 days, she continued to be febrile, and her CSF cultures grew *L. adecarboxylata* with sensitivities seen in Table 1. Initial gram stain showed abundant PMNs with abundant gram-negative rods. Repeat gram stain 4 days later showed abundant PMNs but no organisms. She underwent repeat CSF cultures (Table 2) to ensure bacterial clearance. Based on antibiotic sensitivities (Table 1), her antibiotics were subsequently narrowed to ceftriaxone 2 g IV every 12 h.

She completed a 2-week course of ceftriaxone for *L. adecarboxylata* ventriculitis. Afterward, given persistent intracerebral pressure, she underwent ventriculoperitoneal shunt placement. She was discharged to an acute rehabilitation facility off antibiotic therapy and has been without any evidence of recurrent or persistent infection for 6 months.

## 3. Discussion

This case series describes two cases of *L. adecarboxylata* causing monomicrobial neurosurgical infections. To our knowledge, these are the first two neurosurgical cases of monomicrobial *L. adecarboxylata* infection in immunocompetent hosts. Prior reported cases were in immunocompromised hosts and associated with polymicrobial infections [7]. Zayet et al. published a small case series in 2021, where five out of the six cases were immunocompetent, and four of the six cases were polymicrobial [1]. The infections in this case series were associated with indwelling medical devices, suggesting that *L. adecarboxylata* may become part of the skin flora in hospitalized patients. The exact epidemiologic source of most *L. adecarboxylata* infections reported has not been determined. *L. adecarboxylata* is ubiquitous in the environment, with studies showing that this bacterium is commonly found in soil [8] and is part of the gut microbiome in a variety of animal reservoirs [9]. One study even found it to be a commensal bacterium with the *Anopheles subpictus* mosquito in India, raising the possibility of vector-borne transmission [10]. In some cases, there have been direct epidemiological linkages with environmental exposures, such as a swimming pool, but most are unknown [11]. In our series, neither patient was immunocompromised, had new environmental exposures, nor traveled outside of the United States in the year leading up to their diagnosis. The most likely source of the monomicrobial infection in our two cases is nosocomial acquisition. The leading hypothesis is that, in both patients, their skin flora was altered, changing from predominately gram-positive microbes to gram-negative microbes during their prolonged hospitalization [12]. This may have led *L. adecarboxylata* to cause spinal infection and EVD-associated ventriculitis, given the open draining wound in Case 1 and the crack in the initial EVD in Case 2. Several studies support this hypothesis, showing that individuals can become colonized with *L. adecarboxylata*, causing asymptotic transmission to immunocompromised contacts [5]. Alternatively, a previously healthy asymptomatic carrier can become immunocompromised and become susceptible to active infection [5].

However, why this bacterium was responsible for the neurosurgical infections seen here and not another pathogen is poorly understood. The small number of cases involving this organism makes identifying pertinent risk factors and virulence factors challenging. To note, *L. adecarboxylata* is typically sensitive to numerous antibiotics and has yet to acquire significant antibiotic resistance genes. As seen here, both isolates were sensitive to numerous antibiotics, which is consistent with prior studies [1, 6]. It may be that this pathogen outcompeted other bacteria, given its ability to devote energy to replication and not resistance gene expression. While this is speculative, it does reinforce that more research is warranted to further assess this emerging pathogen and thereby prevent *L. adecarboxylata* infections. Treatment duration is also dependent on the nature of the infection caused. The first case was treated for 6 weeks due to concern for osteomyelitis, given the involvement of the patient's spinous process at L4-L5. The second case was due to an EVD-associated ventriculitis and was treated for 2 weeks after the removal of the device-achieved source control. In both cases, the patients received short courses of antibiotics prior to culturing *L. adecarboxylata*. The ability to recover the organism despite treatment was likely due to poor CNS penetration by the beta-lactams used and a lack of source control, allowing persistent bacterial growth. In particular, for Case 2, while the patient had received 7 days of vancomycin and piperacillin–tazobactam prior to diagnosis, the former agent has no activity against *L. adecarboxylata,* and the latter has poor CNS penetration, likely creating an environment where other organisms were suppressed, allowing the infection to take hold.

This bacterium may be more prevalent in causing infections than previously recognized, and with improved diagnostics, we may be identifying this organism more often. Prior underrecognition was because *L. adecarboxylata* grows similarly to other members of the *Escherichia* genus on culture media including MacConkey agar [13].

The introduction of matrix-assisted laser desorption/ionization time-of-flight mass spectrometry (MALDI-TOF) for the identification of clinical sample isolates appears to have led to more reliable and frequent identification of *L. adecarboxylata* [14]. Subsequently, infection rates appear to be rising, but more research is needed to tease out if this is a true rise in incidence or simply due to improved diagnostics, allowing for better recognition of the pathogen.

In conclusion, this case series presents two cases of neurosurgical infections caused by *L. adecarboxylata.* Both cases reinforce that *L. adecarboxylata* is emerging as a clinically significant pathogen, but it is unknown if the increases in reported cases are secondary to improved diagnostics or if these infections are increasing in incidence. As seen here, most cases of *L. adecarboxylata* have unknown epidemiological sources. This case series highlights *L. adecarboxylata's* potential as a nosocomial pathogen, underscoring the need for heightened clinical awareness and further investigation into its epidemiology to prevent further infections.

## Figures and Tables

**Table 1:** Antibiotic susceptibilities of the two *L. adecarboxylata* described in the case series.

Antibiotic tested	Case 1		Case 2	
	Interpretation	MIC (μg/mL)	Interpretation	MIC (μg/mL)
Amikacin	Sensitive	≤ 2	Sensitive	≤ 16
Ampicillin	Not tested	Not tested	Sensitive	≤ 8
Ampicillin/sulbactam	Sensitive	≤ 2	Sensitive	≤ 4/2
Aztreonam	Sensitive	≤ 1	Sensitive	≤ 4
Cefazolin	Sensitive	≤ 4	Not tested	Not tested
Cefepime	Sensitive	≤ 1	Sensitive	≤ 2
Ceftriaxone	Sensitive	≤ 1	Sensitive	≤ 1
Ciprofloxacin	Not tested	Not tested	Sensitive	≤ 0.25
Ertapenem	Sensitive	≤ 0.5	Not tested	Not tested
Gentamicin	Sensitive	≤ 1	Sensitive	≤ 2
Levofloxacin	Sensitive	≤ 0.12	Sensitive	≤ 0.5
Meropenem	Sensitive	≤ 0.25	Sensitive	≤ 1
Piperacillin/tazobactam	Sensitive	≤ 4	Sensitive	≤ 8
Tobramycin	Not tested	Not tested	Sensitive	≤ 2
Tetracycline	Sensitive	≤ 1	Not tested	Not tested
Tigecycline	Sensitive	≤ 0.5	Not tested	Not tested
Trimethoprim/sulfamethoxazole	Resistant	≥ 320	Sensitive	≤ 0.5/9.5

Abbreviation: MIC, minimum inhibitory concentration.

**Table 2:** Laboratory characteristics of CSF collected from the patient in Case 2.

	Diagnosis of ventriculitis	After 4 days of antibiotic treatment
Glucose (mg/dL) (normal range 40–70)	51	55
Lactate (mmol/L) (normal range < 2.7)	6.3	3.9
Protein (mg/dL) (normal range 12–60)	160	150
White blood cell (cells/mcL) (normal range 0–5)	756	65
PMN (% of WBC)	76	2
Gram stain	Abundant gram-negative rods and PMN	No organisms seen, light PMNs
Culture	*L. adecarboxylata*	No growth after 5 days

*Note:* PMN, polymorphonuclear cell.

Abbreviation: WBC, white blood cell.
